# Clinical Society

**Published:** 1897-01-16

**Authors:** 


### Medical Progress and Hospital Clinics.

[*The Editor will be glad to receive offers of co-operation and contributions from members of the profession. All letters should be addressed to* The Editor, at the Office, 28 & 29, Southampton Street, Strand, London, W.C.]

### CLINICAL SOCIETY.

Mr. Brudenell Carter described a case of gunshot wound of the left eye, which was penetrated by two pellets of snipe shot. The pellets passed through the whole thickness of the lower lid, and pierced the eyeball below the cornea. Assuming them to have been sterilised by the heat of the discharge, or even mechanically by their passage through the lid, Mr. Carter thought that nothing more than the physical effects of the injury need be anticipated, and pursued an expectant treatment. Blood effused within the eye was gradually absorbed, and the pellets could then be seen adherent to the inner surface of the sclera and encapsuled by lymph. The retina and choroid had been divided, and had retracted, leaving a large white patch; but central vision equal to ten-twelfths of the normal was recovered. A drawing of the injury and of the pellets was exhibited. In the discussion which followed, the author strongly condemned the practice of enucleation in cases of injury, and maintained that Mules's operation should always be preferred.

Dr. Herringham related an interesting case of paroxysmal tachycardia in a child eleven years of age. She had had seven attacks in the course of ten months. She was in good health, and the attacks could not be traced to temporary disturbance of function of any organ. The attacks lasted a variable time, from a day and a-half to thirteen days; they began suddenly, and always ended in the night; the palpitation did not prevent sleep, and it went on during sleep; usually the attacks seemed to follow some unaccustomed movement, but this could not always be verified. There was no cardiac bruit, but the heart was somewhat enlarged permanently, and became still more dilated during the attacks. There were no signs of high tension in the pulse; in fact, the radials were peculiarly small, and amyl nitrite did not afford any relief. During the paroxysm the skin became a little dusky, and, if it continued for a long time, slightly yellow. The pulse was sometimes uncountable at the wrist, but at the heart had been counted up to 256 in the minute. These attacks had been going on for five or six years. Dr. Herringham had searched the literature, but could only find one other case in a child described, although in several adult cases it was noted that the disease had continued since childhood.

In the very interesting discussion which followed, it was pointed out that these cases of paroxysmal heart-hurry were to be placed in quite a different category from those in which, as a symptom of such a condition as Graves's disease, the pulse was quick; and the term paroxysmal tachycardia, used by Dr. Herringham, seemed properly to express the most striking features of the condition in question. Notwithstanding the fact that between the attacks the patients appeared in good health, the condition must always be looked on as a grave one, for a considerable proportion of the patients in whom it occurred ultimately died with cardiac symptoms. Although the late Dr. Bristowe had expressed the opinion that these attacks occurred in healthy hearts, yet some even of his cases had ended as cardiac affections.

One speaker stated that he had seen four or five cases in adults, all of whom had died, and he pointed out that in each of these cases there was disease or dislocation of some abdominal organ, such as the uterus or kidney. Dr. Herringham, however, thought that this could not be looked on as an essential cause, seeing that in one of the best noted cases of this group, in which the attacks were associated with a movable kidney, they had commenced quite in childhood, when we could hardly expect the kidney to be in that condition. Attention was drawn to the dilatation of the heart and the remarkable murmurs which were sometimes developed in the course of the attacks; and Dr. West related the case of a policeman, in whom, although the palpitation produced no particular distress, it was accompanied with a loud and extraordinary bruit that it could be heard by the man in the next bed. In this case also great dilatation was produced in each attack, but after recurring frequently for a couple of years the disease passed off, leaving him perfectly sound, and he had remained so for the past six or seven years. Nevertheless, he did not look on the prognosis as anything but serious in such cases; for, although cure sometimes occurred, in other cases the liability to the attacks lasted for the whole life, and in the majority of the cases severe cardiac symptoms ultimately developed.

The therapeutic suggestions thrown out were, the use of arsenic, valerianate of zinc, and bromide of ammonium; and, during the course of the attack, compression of the chest, by squeezing the thorax and abdomen, which had been found by Rosenfelt to be effectual in cutting short the paroxysms.

Mr. C. S. Wallace showed a specimen of fracture of the axis which tended to upset completely the assertion so commonly made as to the necessarily fatal result of fracture of the upper spinal vertebræ. The patient fell down an area, a distance of nine feet, but exact particulars as to the nature and direction of the violence could not be obtained. He was put to bed by his friends, and next day travelled to the hospital in a tramcar, and he walked into the surgery without supporting his head, which, however, was fixed with the face looking downwards, the chin being almost in contact with the sternum. No displacement of any vertebræ could at the time be felt. He was put to bed, and he gradually improved to such an extent that he could rotate his head from side to side, could flex and extend it, and could raise it from the pillow. There was no paralysis at any time. On the eighth day, however, while the nurse was changing the draw-sheet, the patient's breathing suddenly showed signs of embarrassment; he became cyanosed, and died. The specimen showed a fracture of the axis, by which the odontoid process and the left superior articular facet were completely separated from the rest of the bone by a fracture running downwards and forwards. The transverse ligament was untorn. It seemed evident that but for an untoward accident this patient might have recovered, notwithstanding the complete fracture of this important vertebræ.

